# Optimization of Case Finding and Preventive Treatment Among Household Contacts of People with Tuberculosis in Zimbabwe

**DOI:** 10.3390/tropicalmed10120347

**Published:** 2025-12-10

**Authors:** Tawanda Mapuranga, Collins Timire, Ronald T. Ncube, Sithabiso Dube, Nqobile Mlilo, Cynthia Chiteve, Owen Mugurungi, Fungai Kavenga, Manners Ncube, Nicholas Siziba, Selma Dar Berger, Talent Maphosa, Macarthur Charles, Julia Ershova, Riitta A. Dlodlo

**Affiliations:** 1 The Union Zimbabwe Trust, 261 Diamond Road, Westgate, Harare P.O. Box CY 550, Zimbabwe; rncube@uzt.org.zw (R.T.N.); sdube@uzt.org.zw (S.D.); nmlilo@uzt.org.zw (N.M.); cchiteve@uzt.org.zw (C.C.); 2 AIDS and TB Department, Ministry of Health and Child Care, Causeway, Harare P.O. Box CY 1122, Zimbabwe; collinstimire2005@yahoo.com (C.T.); mugurungi@gmail.com (O.M.); drkav8@gmail.com (F.K.); ncube.manners@gmail.com (M.N.); sitwalo@gmail.com (N.S.); 3 The International Union Against Tuberculosis and Lung Disease (The Union), 75001 Paris, France; selmadar@gmail.com (S.D.B.); rdlodlo@theunion.org (R.A.D.); 4 US Centers for Disease Control and Prevention, Harare 00263, Zimbabwe; oxv9@cdc.gov; 5 US Centers for Disease Control and Prevention, 1600 Clifton Road, Atlanta, GA 30329, USA; xzk9@cdc.gov (M.C.); jhe3@cdc.gov (J.E.)

**Keywords:** close contacts, TB preventive treatment, contact investigation, contact tracing, Zimbabwe

## Abstract

Systematic screening of household contacts (HHCs) of people with tuberculosis (TB) and starting them on either TB treatment or tuberculosis preventive treatment (TPT) reduces TB incidence. This project supported HHC management in six health facilities in Zimbabwe through the provision of CXR services, reimbursement of transport costs for HHCs, and provision of fuel and refreshments for healthcare workers involved in contact tracing. We describe TB and TPT cascades among the HHCs of index patients with all forms of TB. We enrolled 251 index patients who listed 794 HHCs: 551 (69%) HHCs of 158 index patients were traced and 520 (94%) screened for TB. Of the 502 who were referred to clinics, 362 (72%) reached the clinic. Among 520 HHCs, 324 (62%) underwent CXR screening and 18 (5%) had CXRs suggestive of TB. The yield of TB was 2.3% (12/520), with CXR detecting eight people who had not reported TB symptoms. Of the 311 who were assessed for TPT eligibility, 126 (41%) started TPT and 119 were assessed for TPT outcomes. Of these, 111 (93%) had successful TPT outcomes. The median times to starting TB treatment and TPT were 7 days and 11 days, respectively. The intervention facilitated timely access to healthcare services and a high yield of TB detection.

## 1. Introduction

Almost a quarter of the global population has tuberculosis (TB) infection (TBI). If left untreated, people with TBI have a 5–10% lifetime risk of progressing to active TB disease [1,2,3]. In low- and middle-income countries (LMICs), approximately 2.2% to 4.4% of contacts of people with TB have TB disease themselves; the incidence of TB in contacts is highest in the first year after exposure, especially among children under 5 years and people living with HIV (PLHIV) [4,5].

Historically, TB control efforts have focused on diagnosing and treating TB disease. This has changed since the launch of the End TB strategy, which targets a 90% reduction in global TB incidence by 2035 compared to the 2015 baseline [1,6,7]. This strategy seeks to reduce TB incidence through interventions along the whole care cascade from TB prevention to early detection and treatment, including TB preventive treatment (TPT), which greatly reduces the risk of progression from TBI to TB disease [5]. Early TB diagnosis and treatment reduces community transmission and maximizes health outcomes, ensuring people do not develop advanced TB and minimizing the loss of productive capacity, a major driver of catastrophic costs [8]. TPT reduces reservoirs of TBI, thereby reducing TB incidence [9].

Systematic screening of contacts of TB patients for TB and TBI and improving coverage of TPT are priorities of the End TB strategy to eliminate TB [5]. Household contacts (HHCs) of TB patients have a heightened risk of TBI as well as progression from TBI to TB disease, owing to household members sharing risk factors for TB such as malnutrition and crowded living conditions [10]. While malnutrition increases susceptibility to TBI and advanced TB disease [11,12,13,14], crowded spaces increase the duration and intensity of exposure to TB by mycobacteria from household members with TB, especially when TB treatment is delayed [15]. For this reason, TPT is recommended for the HHCs of people with bacteriologically confirmed pulmonary TB [6,16]. World leaders at the United Nations High-Level Meeting (UNHLM) in 2023 pledged to provide TPT to 30 million HHCs (all age groups) for the period 2023–2027. Although TPT initiations among HHCs have improved since 2015, coverage remains suboptimal at ~25%, well below the target of 90% by 2027 [6,17,18].

Standard household contact tracing approaches rely on screening contacts in communities and referring them to health facilities for TB investigations and TPT. However, contacts usually have little incentive to seek evaluation for conditions they do not perceive themselves to have [19]. especially if there are barriers to accessing such services. Consequently, up to 40% of contacts who are referred for TB investigations and TPT initiation fail to reach or delay reaching health facilities by up to 2–3 months [20]. Screening of contacts is usually performed using a symptom screen, which is less sensitive than CXR. Consequently, the yield of active case finding is low and asymptomatic TB cases are frequently missed [21].

Zimbabwe as a country has a high TB burden, with an estimated TB incidence of 203 per 100 000 population in 2024 [22]. The national TB guidelines recommend contact tracing and TB screening among all contacts of people diagnosed with pulmonary TB (all forms and all ages) [22]. TB screening is conducted using the WHO four-symptom screening tool (W4SS). Those responding yes to any of the four symptoms are presumed to have TB, and further investigation must be performed to confirm active TB. Chest X-ray, when available, is used as a screening tool for high-risk groups like people living with HIV (PLHIV), health workers, TB contacts, and prisoners. For a TB diagnosis to be confirmed, a sputum sample is often collected for testing using WHO-recommended molecular tests like GeneXpert. For children, among whom sputum is often difficult to collect, a stool sample is used. However, HHC tracing is usually associated with marked losses along the care cascade [23,24]. The TPT coverage among the HHCs of bacteriologically confirmed cases in Zimbabwe was insufficient at the start of the project, namely 41% in 2023 [25]. Innovative approaches to minimize losses along the care cascade must optimize the screening of contacts within communities, ensuring they reach health facilities for TB investigation and TPT initiation.

To address this gap, we undertook an HHC investigation project in six facilities in Zimbabwe that receive support for TB and HIV care from the United States President’s Emergency Plan for AIDS Relief (PEPFAR).

The project aimed to reduce barriers to TB screening and diagnostic services for HHCs through targeted interventions designed to optimize and improve access and efficiency. The primary objectives were to optimize the initiation of TB treatment and TPT in intervention facilities by scaling up globally recommended guidelines adapted to the national context, using a comprehensive patient-centered approach that included radiological screening of TB contacts. Additionally, the study aimed to describe the TB and TPT care cascades for household contacts of TB patients across all age groups to identify gaps in implementation. Secondary objectives included strengthening healthcare worker capacity to improve TPT delivery, ensuring accurate documentation of high-quality data, and promoting local data analysis to inform evidence-based decision-making.

Here we describe the TB and TPT cascades among HHCs and assess the yield of TB among HHCs.

## 2. Methods

### 2.1. Site Selection

This prospective program evaluation was conducted in Mashonaland East, Central, and West provinces. Each of the three provinces was represented by two PEPFAR-supported facilities with differences in level and type of care (provincial, district, and mission hospitals, as well as one clinic). Four facilities had radiology services; project participants from two other facilities were referred for CXR to facilities close by, with radiology service available. Table 1 summarizes the 2021 notification data and other characteristics for the six selected sites.

### 2.2. TB Contact Investigation Approach

According to national guidelines, contact investigations are carried out for index cases with bacteriologically confirmed and clinically diagnosed pulmonary TB, any form of pediatric TB irrespective of location or bacteriologic status, and multidrug-resistant TB (MDR-TB) [24]. Two models for contact tracing exist: facility-based, where contacts opt to come to the health facility, and community-based, where environmental health technicians (EHTs) or community health workers (CHWs) conduct the tracing activities at the patients’ home. TB contacts who are eligible for TPT are referred to a health facility to initiate treatment and are usually given a full TPT course to self-administer at home.

The project provided a comprehensive person-centered approach that differed from the standard of care and was optimized to increase the detection of TB disease among HHCs and improve access to TPT eligibility assessments. Interventions included routine CXR screening for all identified contacts regardless of the presence of symptoms suggestive of TB; financial assistance for contacts unable to afford CXR fees; reimbursement of transport costs for contacts to access health facilities; provision of enablers for EHTs and CHWs (e.g., face masks, sanitizers, refreshments, fuel for home visits’ transportation); training of HCWs on TPT; and distribution of adapted contact investigation registers and tracing forms, as well as airtime for HCWs to ensure prompt communication. Furthermore, in addition to the existing TPT regimens for eligible adult contacts, a one-month regimen of combined isoniazid and rifapentine (1HP) was introduced at the six selected project sites. This intervention package aimed to improve the detection of TB disease among household contacts of index patients with bacteriologically confirmed or clinically diagnosed pulmonary TB, facilitate initiation of TPT for eligible contacts, and strengthen access, adherence, and completion of TPT within intervention facilities.

### 2.3. Contact Elicitation and Home Visits

Household contacts of all index patients with newly diagnosed pulmonary TB (bacteriologically confirmed and clinically diagnosed by clinical judgement with a negative microbiologic confirmation), household contacts of children < 5 years of age newly diagnosed with any form of TB (reverse contact investigation), and contacts of persons with DRTB irrespective of the HIV status of the index patients were included. Index patients were identified in the health facility TB registers as they were recorded in each facility. Each index TB patient (pulmonary bacteriologically confirmed, pulmonary clinically diagnosed, multi drug-resistant TB, or childhood TB) identified in the selected facilities was interviewed by an HCW in the facility about close contacts as per the national guidelines [26]. Those who had contacts were asked to provide the name, sex, and age of the contacts and location information (physical address and phone number) which were then listed in the contact tracing form and registered against each index case. Index cases were offered a home visit by HCWs for the purposes of contact investigation. At the home, the EHT assessed and educated the household members about infection prevention and control and then screened all contacts using a symptom screening tool. Irrespective of the screening outcome, the EHT referred all contacts to the catchment health facility for further evaluation for TB and TPT eligibility. The EHT also informed them of free CXR services and transport reimbursement. When the EHT was not able to carry out a household visit, a trained CHW from the index patient’s catchment area was assigned to carry out contact tracing.

### 2.4. Contact Investigations at Health Facility

At the health facility, the EHT recorded the outcome of each home-visit screening activity in the contact tracing register. Contacts who visited the health facility were fast-tracked and given a CXR coupon for a radiology center which was either located within the health facility or subcontracted by the project to a private service provider. Digital, non-AI-based CXRs were interpreted either by a specialist radiologist (for subcontracted services) or by the medical officer on duty (for onsite radiology services). These clinicians had received training through pre-service and institutional programs, supplemented by TB-ECHO sessions, and were supported by institutional physicians for quality assurance. The contact tracing register captured symptom screening results, CXR findings, and whether or not the contact was presumed to have TB, based on (i) a positive symptom screening only (for those who did not access CXR), (ii) positive symptom screening and CXR suggestive of TB (interpreted independently by clinician as abnormal with findings pointing towards the possibility of TB), or (iii) no symptoms but CXR suggestive of TB.

### 2.5. Laboratory Investigations for Contacts with Presumptive TB

The names of all HHCs who were presumptive for TB were recorded in the presumptive TB register and asked to submit a spot sputum specimen (or stool for children) for testing using the Xpert MTB/RIF Ultra assay (Cepheid, Sunnyvale, CA, USA). Those who tested positive were recorded in the TB treatment register and started on TB treatment according to the national TB guidelines.

### 2.6. TPT Eligibility Assessments and Initiation

All HHCs who were not presumptive for TB and in whom TB was ruled out by molecular tests were assessed for TPT eligibility at the health facility. Those eligible were recorded in the TPT register and started on an appropriate TPT regimen based on Zimbabwe’s national TB guidelines [25]. Following eligibility assessment, child HHCs under 15 years who were HIV-negative and in whom active TB was excluded were offered either a 3-month regimen of isoniazid and rifampicin (3RH) or, alternatively, a 6-month isoniazid regimen (6H), after appropriate counselling and parental/guardian consent. Adult HHCs (≥15 years) who were HIV-negative and free of active TB were offered 3HP (isoniazid plus rifapentine for 3 months) or 6H. As part of the project-specific intervention, a 1-month regimen of isoniazid and rifapentine (1HP) was also provided to eligible adults, subject to drug availability. Additionally, HHCs diagnosed with HIV received TPT according to the national guidelines for HIV-positive clients: 3HP for those aged ≥ 2 years, and 6H for those under 2 years. For children living with HIV (CLHIV) aged 2–14 years who were concurrently receiving dolutegravir-based antiretroviral therapy, 3HP was avoided and replaced with 6H to prevent drug–drug interactions.

### 2.7. Payment of CXR Services, Transport Reimbursements, and Enablers

The project engaged administrators at each implementing health facility to reconcile cash for transport reimbursement. Funds for CXRs were paid directly to service providers through an arranged transfer system after reconciliation of total costs incurred and verified through the CXR coupon booklet. Enablers for EHTs and CHWs were reconciled and paid based on the field activities conducted.

### 2.8. Data Collection

Quantitative data for index patients and their household contacts were collected using standardized questionnaires. The following data were collected for the purposes of this project.

Index patients: Age, sex, type of TB (pulmonary bacteriologically confirmed, pulmonary clinically diagnosed, extra-pulmonary TB, multidrug-resistant TB (MDR-TB), TB treatment initiation, interview about contacts, home visit offer, location (local catchment area/outside local catchment area), and number of contacts listed.

Contacts: Age, sex, HIV status, type of contact, relationship to index patient, whether or not screened for TB, cadre responsible for screening, TB symptoms elicited, whether contact was referred and reached referral facility, referral, and results for CXR, and whether TB was presumed (Y/N).

For those not presumptive for TB or had TB ruled out, TPT-related data were collected including whether the contact was assessed for TPT eligibility, registration date, and date when TPT was initiated, TPT regimen, and outcome of TPT. People who either completed TPT (finished 1 month of 1HP or 3 months of 3HP/3RH or 6 months of INH course of TPT without evidence of failure or stopping due to adverse events) or were still on treatment or were transferred out whilst on TPT were considered to have successful TPT outcomes. Those who died or whose outcomes were not recorded were considered to have unsuccessful TPT outcomes. For those presumptive for TB, data were collected on the date of registration in the presumptive register, sputum collection status and transmission to laboratory, and laboratory results. For those diagnosed with TB, data were collected on the type of TB and date of treatment initiation.

Data collection was carried out during two scheduled visits. All data were entered into a relational database created in the EpiCollect5 application (https://five.epicollect.net (accessed on 20 July 2023)), installed on android-based tablets procured for the purposes of the project. Data were uploaded to a secure cloud-based server. Periodic data query logs were run as a Stata do file to check for completeness and consistency. All variables with data queries were listed by IDs, health facility, and ID of data entry personnel so that corrections could be made. All incomplete data identified during the first data collection visit were updated during the second data collection visit.

### 2.9. Data Analysis

Data were exported to Stata version 13.0 (StataCorp, College Station, TX, USA) and Epi Info version 7.2.6.0. (Epi Info™, CDC, Atlanta, GA, USA) for cleaning and analysis. Categorical variables were analyzed using numbers and proportions. The results were presented as TB and TPT cascades. Continuous variables were analyzed using means and standard deviations (SD) for normally distributed data or medians and interquartile ranges (IQRs) for skewed data.

### 2.10. Ethics

Ethics approval was obtained from the Medical Research Council of Zimbabwe (approval number MRCZ/A/2746). This activity was reviewed by CDC, deemed not research, and conducted in accordance with the applicable federal law and CDC policy. Data confidentiality and anonymity were ensured through the use of unique IDs and data were secured using a password protected cloud-based server that complies with General Data Protection Regulations (GDPR) UK.

## 3. Results

A total of 251 newly diagnosed index patients were enrolled in the project from 14 November 2023 to 31 July 2024. The characteristics of index TB patients are shown in Table 2: the mean age was 39.6 years (SD = 18.6), 164 (65%) were men, and almost 50% had bacteriologically confirmed pulmonary TB. Overall, 244 (97%) initiated TB treatment. Contact listing involved documenting all the HHCs as mentioned by the index case during the interview with the clinician at the health facility; tracing involved following up these enlisted contacts during a scheduled home visit by the EHT or CHW; screening consisted of face-to-face symptom-screening interviews administered to HHCs present during the home visit or to those who subsequently presented at the health facility. Clinical diagnosis was made by clinicians using symptom evaluation, physical examinations, and CXR abnormalities when microbiological confirmation was not available. Of the 243 index TB patients who were interviewed about HHCs, 204 (84%) listed at least one contact. Overall, 794 HHCs were listed; the median number of HHCs per index patient was 3 (IQR: 1–5), ranging from 1 to 13.

Of the 794 listed HHCs, 551 (69%) HHCs from 157 index TB patients were traced. The mean age of the traced HHCs was 25.6 (SD = 18.5) years, and almost 50% were men. Approximately one-third of the HHCs were children under 15 years (Table 3).

### 3.1. TB Investigations Among HHCs

**Figure 1:**
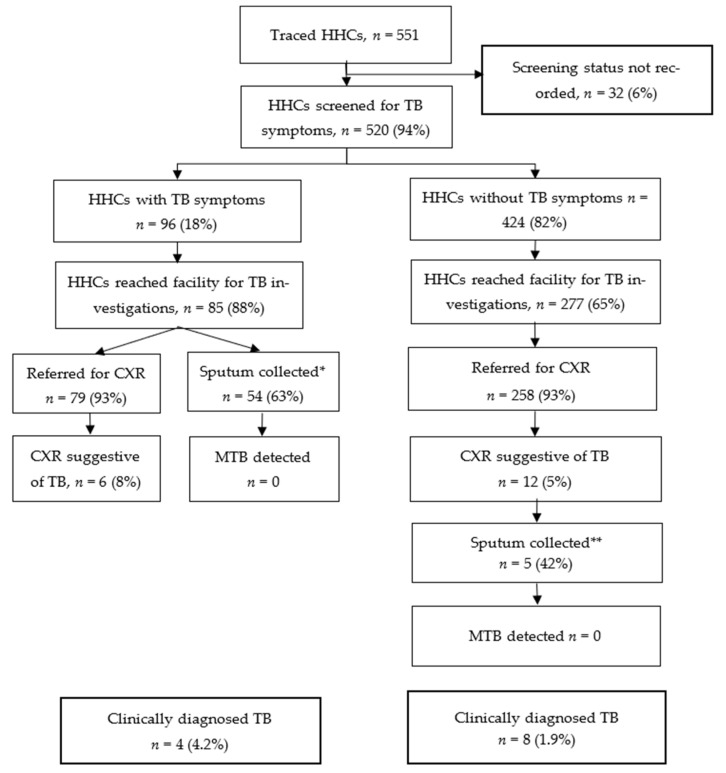
TB cascade among traced HHCs from 157 TB index patients at six health facilities in Zimbabwe—14 November 2023 to 31 July 2024 (*n* = 551); * For 31 HHCs, sputum was not collected; this was mainly due to failure to produce sputum by clients, clients not being patient for sputum collection procedure, intermittent non-availability of sputum containers, or collection was not recorded. ** For 7 HHCs, sputum was not collected; this was mainly due to failure to produce sputum among asymptomatic HHCs or collection was not recorded.

Of the 551 traced HHCs, 520 (94%) were screened for TB symptoms; 96 (18%) reported at least one TB symptom. Among the 96 who had TB symptoms, 85 (88%) reached health facilities for TB investigations and 79 (93%) had CXR investigations. Of the 424 (82%) HHCs who did not report TB symptoms, 406 (96%) were referred to health facilities for TB investigations; 277 (68%) reached facilities and 258 (93%) were referred for CXR investigations. A total of 324/520 (62%) HHCs underwent CXR screening and 18 (5%) had CXRs suggestive of TB. Twelve (5%) HHCs had presumptive TB based on CXR (Figure 1).

Overall, 108 HHCs had presumptive TB, including 96 based on TB symptoms and 12 based on CXR results suggestive of TB. Of these, 59 (55%) had their sputum collected for laboratory testing; MTB was not detected in any specimens. In total, 12 HHCs (8 males and 4 females) were diagnosed clinically with TB, resulting in a yield of 2.3% among the 520 screened HHCs. Two HHCs diagnosed with TB were children <15 years old. A total of 8 (67%) of the 12 HHCs diagnosed with TB were asymptomatic and initially identified by CXR; they would have been missed by symptom screening alone. Seven clients diagnosed with TB were among the contacts of 71 index patients with clinically diagnosed pulmonary TB, while five clients were contacts of 74 index patients who had pulmonary bacteriologically confirmed TB. In terms of the number needed to screen based on the index patient’s status and irrespective of the number of HHCs per index case, it took an average of 10.1 (71/7) HHC screenings from clinically diagnosed index cases to identify one contact with TB, and 14.8 (74/5) HHC screenings from bacteriologically confirmed index cases to find one contact with TB.

### 3.2. TPT Initiation Among HHCs

**Figure 2:**
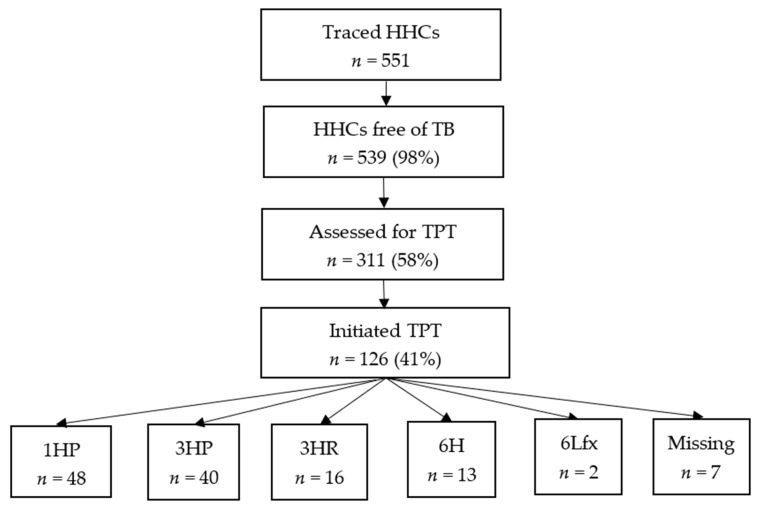
TPT cascade among traced HHCs from 157 TB index patients at six health facilities in Zimbabwe—14 November 2023 to 31 July 2024 (*n* = 551); 1HP = 1 month of daily isoniazid and rifapentine; 3HP = 3 months of weekly isoniazid and rifapentine; 3HR = 3 months of daily isoniazid and rifampin; 6H = 6 months of daily isoniazid; 6Lfx = levofloxacin = 6 months of daily levofloxacin.

Of the 539 HHCs that were not diagnosed with TB, 311 (58%) were assessed for TPT eligibility: 126 (41%) started TPT (limited coverage mainly due to intermittent stock-outs of TPT medicines). Of the 119 who were assessed for TPT outcomes, 111 (93%) had successful outcomes (Figure 2, Table 4). Among the 126 HHCs who started TPT, 61 (48%) were males and 48 (38%) were <15 years old. In total, 10 (8%) HHCs were HIV-positive, 39 (31%) were HIV-negative, and 79 (61%) did not have a documented HIV status at the time of TPT initiation.

### 3.3. Timelines for TB and TPT Activities Among HHCs

Of the 236 index patients who had valid dates regarding TB diagnosis and the listing of HHCs, 98% of their HHCs were listed within 7 days (Table 5). The median time from HHC listing to a HHC reaching the health facility was 6 days (IQR = 3–25), with close to 60% of HHCs reaching the health facility within 7 days after the listing. All 12 contacts found to have TB started TB treatment, and 8 of them initiated treatment within 7 days of TB diagnosis. The median time from the listing of HHCs to starting TPT was 11 days (3–28) and almost 50% started TPT within 7 days.

## 4. Discussion

We sought to describe the TB and TPT cascades among the HHCs of index TB patients, including those who were bacteriologically confirmed and those clinically diagnosed. Although the yield of TB detection was high, TPT initiation among HHCs in whom TB was excluded remained low. Notably, HHCs of clinically diagnosed pulmonary TB patients were just as likely to be diagnosed with TB as contacts of bacteriologically confirmed patients. A high proportion of HHCs reached health facilities and accessed CXR services, but none of them had bacteriologically confirmed TB, even among those with CXRs suggestive of TB. Household contact tracing activities were delivered in a timely manner with 50% of TB and TPT initiations conducted in under 7 days.

The yield of 2.3% among HHCs is higher than the population incidence in Zimbabwe, substantially exceeding what would be expected—203 per 100,000 population [27]. The yield of TB observed in our project is comparable to yields reported from several LMICs, including Uganda, Brazil, and Pakistan [28,29,30]. Overcrowding and prolonged exposure (≥6 h per day) of index TB patients to HHCs could increase both the duration and intensity of exposure to MTB infection among HHCs [31]. One might expect higher yields among HHCs of index patients with bacteriologically confirmed pulmonary TB because they are likely to have lung cavities and a higher risk of transmitting MTB infection to their contacts [32]. However, our findings did not reflect this expectation. Rather, our results confirmed findings from studies that reported similar yields of TB among the HHCs of index patients with clinically diagnosed and bacteriologically confirmed TB [33,34]. Our yield of TB was lower than yields reported from South Africa, Uganda, and Pakistan, possibly due to the higher incidence of TB in these countries or spatial variation within the countries [35,36,37,38].

None of the HHCs diagnosed with TB in our study were bacteriologically confirmed, suggesting that we may have identified individuals early in the progression of the disease. In our study, 67% of those diagnosed with TB were asymptomatic, indicating that relying solely on symptom screening would have resulted in missing a significant portion of cases. Without appropriate treatment, approximately 10% of adults with CXRs suggestive of TB progress from microbiologically negative to bacteriologically confirmed TB within a year [39]. Some studies have successfully increased yield by conducting follow-up tests among HHCs who were microbiologically negative at baseline, a strategy that was not employed in our study [40].

Access to health facilities for TB and TPT investigations is crucial to ensure early diagnosis and care. In our project, the majority of HHCs reached health facilities for TB investigation; this may be at least partially due to travel reimbursements that were provided by the project. Contacts, just like people with TB, are also affected by socioeconomic and structural barriers to accessing TB and TPT services. Across similar settings, HHC tracing activities experience considerable losses along the cascade of care due to several barriers [23,24]. The barriers range from structural barriers (long distance to health facilities and transport costs), health system barriers (lack of experience among HCWs to prescribe TPT [41] inflexible opening hours, long waiting times, lack of TPT medicines, and lack of sensitive TB screening tools to rule out active TB) [39,40]. and patient-related barriers such as opportunity costs of visiting health facilities, especially among relatively healthy cohorts who do not perceive themselves to be ill [42,43,44]. People who do not have TB symptoms are less likely to feel that they may benefit from screening compared to those with symptoms, as evidenced by our finding of the higher proportion of HHCs who reported TB symptoms who reached health facilities compared to those who did not. Optimal linkage to care among HHCs is ensured when barriers to accessing healthcare services are alleviated.

The high proportion of contacts who underwent CXR examinations underscores the potential benefits that accrue for communities when barriers to accessing CXRs are removed. Access to CXRs did not vary by symptom status, implying effective linkage to healthcare within health facilities that participated in the intervention. While CXR is a more sensitive TB screening tool than symptom screening [39,40] its access is challenging in most LMICs due to cost and machine downtimes as a result of breakdowns and power outages [34,45]. People are usually referred to private facilities where the cost is usually prohibitive and ranges from USD 10 to 25 in our setting.

Low rates of assessment of TPT eligibility and TPT initiations represented missed opportunities to treat TBI in this project. Several reasons for low uptake have been described in similar studies, including a lack of adequate information regarding benefits and risks of TPT among HCWs, fear of side effects and misconceptions regarding TPT among beneficiaries and caregivers, pill burden, and stock-outs of TPT medicines [46,47,48]. During the implementation period, stock-outs of TPT medicines were experienced nationwide and affected TPT uptake. The frequent stock-outs of TPT medicines could demotivate HCWs to assess TPT eligibility and counsel patients to start TPT when full TPT courses are not available. Lack of adequate knowledge about TPT among HCWs and gaps in the NTP registers that did not include any information on TPT eligibility and reasons for not starting TPT could also have affected the rates of TPT assessment in our project. Well-designed supply aspects (TPT regimens, re-designing of comprehensive registers, and targeted training to improve HCWs’ skills) and demand aspects (education of people and communities about the risks and benefits of TPT) can significantly contribute to effective contact management.

Over time, HHC management has incorporated systematic monitoring of the interval from the initial contact visit to the initiation of TB treatment or TPT, guided by metrics such as the 7-1-7 framework [49]. This framework, which has been adapted for HHC tracing [20,50], recommends that all contacts be (i) listed within seven days, (ii) screened within one day, and (iii) started on TB treatment or TPT within seven days of screening. Previous studies have reported substantial delays, including up to 31 days from index patient treatment initiation to contact screening [51], and TPT initiations occurring more than 60 days later [52]. In our project, nearly all contacts were listed within one week and TB treatment was initiated within the recommended timeframe, although the initiation of TPT was frequently delayed.

This project was conducted in a routine program setting to reflect lived realities as much as possible. However, our intervention provided some additional benefits to the participating facilities and HHCs. We developed a contact TB register to ensure HHCs were line-listed and key variables captured. We engaged radiologists to read CXRs in two facilities, reimbursed HHCs for transportation to conduct CXR, and provided fuel and refreshments for contact investigation teams. Although the supplemental support provided to clinic teams and HHCs was instrumental in achieving a successful intervention, replicating these results in routine settings without comparable resources may be difficult. Nevertheless, current initiatives by the MoHCC to expand digital radiography access, strengthen the hybrid contact investigation model through integrated specimen-transport systems, and explore the task-shifting of community-based TPT initiation to EHTs may improve scalability and programmatic sustainability. Despite the additional support and travel reimbursement, one-third of listed HHCs did not reach health facilities for TB investigation. Furthermore, nearly half of the presumptive TB cases who did present to facilities did not submit sputum specimens, and among those who did, almost one-third lacked laboratory results. These gaps represent missed opportunities for TB diagnosis, and HHCs with incomplete evaluations may continue to have undetected TB, contributing to ongoing community transmission. These findings highlight persistent gaps in the HHC investigation process—likely stemming from limited human and financial resources—and underscore the need for further optimization of person-centered strategies. Our project had several limitations. Information on TPT eligibility and reasons for not starting TPT was not available during data collection. We experienced stock-outs of TPT medicines during the implementation period, which could be a reason for not starting TPT by some HHCs. The project was conducted in facilities that were supported by PEPFAR and provided incentives such as transport, fuel, and refreshments. Hence, the results may not be generalizable to the rest of the country.

Despite these limitations, our project offers several important policy implications. First, it reinforces national guidelines recommending that contact investigations be conducted for contacts of index patients with pulmonary TB, regardless of whether the index case is clinically diagnosed or bacteriologically confirmed. Broadening the scope of contact investigations increases the number of contacts reached and enhances the likelihood of identifying TB that might otherwise remain undetected [52]. Second, our findings highlight the critical role of CXR as a screening tool for early TB diagnosis, particularly for asymptomatic disease. Early identification and timely treatment reduce TB incidence, interrupt community transmission, lower mortality, and mitigate catastrophic costs for affected households. Children—who are highly exposed to infectious caregivers and may develop TB within 90 days of exposure—show higher diagnostic yields than adults [34,38] Their inability to expectorate sputum further underscores the value of CXR screening. National TB programs may therefore consider ensuring free access to CXR. Finally, clearer programmatic guidance is needed for individuals with CXRs suggestive of TB but without bacteriological confirmation, to balance the risks of over-treatment using full TB regimens with those of under-treatment using TPT alone. Esmail et al. have proposed shorter, less intensive regimens for such cases [53]. Further research on this issue is needed.

## 5. Conclusions

The optimized HHC investigation approach resulted in high TB detection yields and improved timely access to essential healthcare services, including chest radiography, TB treatment, and TPT. Strengthening all aspects of HHC management, especially by addressing health system barriers contributing to low TPT initiation—has the potential to reduce losses along the care cascade and enhance community-level screening, thereby ensuring that contacts reach health facilities for comprehensive TB assessment and TPT initiation.

## Figures and Tables

**Table 1:** Selected facilities for household contact tracing intervention in three provinces of Zimbabwe with 2021 TB notification data * and inventories.

Province	Facility	Total TB Cases	%PBC ** Cases	%HIV+ TB Cases	CXR Availability	GeneXpert Availability
Mashonaland Central	Concession district hospital	80	63%	59%	Yes	Yes
	Howard mission hospital	39	23%	54%	Yes	Yes
Mashonaland East	Ruwa rehabilitation hospital	64	87%	45%	Yes (Not functional)	Yes
	Marondera provincial hospital	157	16%	70%	Yes	Yes
Mashonaland West	Katanga Utano clinic	5	100%	84%	No	No
	Chinhoyi provincial hospital	193	19%	46%	Yes	Yes

* 2021 TB notification data as per the national District Health Information Software (DHIS2) system; ** PBC—pulmonary bacteriologically confirmed.

**Table 2:** Sociodemographic and clinical characteristics of TB index patients at six health facilities in Zimbabwe—14 November 2023 to 31 July 2024 (*n* = 251).

Characteristic		*n*	(%)
Sex	Male	164	(65)
	Female	86	(35)
	Not recorded	1	(<1)
Age category	<5	16	(6)
	5–14	7	(3)
	15–24	16	(6)
	25–34	49	(20)
	35–44	76	(30)
	45+	84	(34)
	Not recorded	3	(1)
Facility	Concession district hospital	80	(32)
	Marondera provincial hospital	73	(29)
	Chinhoyi provincial hospital	41	(16)
	Ruwa rehabilitation hospital	29	(12)
	Howard hospital	20	(8)
	Katanga Utano clinic	8	(3)
Type of TB	Pulmonary bacteriologically confirmed	113	(45)
	Pulmonary clinically diagnosed	121	(48)
	Extra-pulmonary	11	(4)
	Drug-resistant pulmonary	4	(2)
	Not recorded	2	(1)
Started TB treatment	Yes	244	(97)
	No	4	(2)
	Not recorded	3	(1)
Location in relation to health facility	Local catchment area of the facility	164	(65)
	Outside catchment area	74	(30)
	Not recorded	13	(5)
Home visit offered to patient	Yes	209	(83)
	No	17	(7)
	Not recorded	25	(10)
Household visited	Yes	158	(63)
	No	93	(37)
Interviewed about contacts	Yes	243	(97)
	No	2	(1)
	Not recorded	6	(2)
Listed any contact	Yes	204	(84)
(among those interviewed, *n* = 243)	No	39	(16)
Index patients with at least one HHC traced	Yes	157	(63)
	No	94	(37)

**Table 3:** Sociodemographic and clinical characteristics of traced HHCs from 157 TB index patients at six health facilities in Zimbabwe—14 November 2023 to 31 July 2024 (*n* = 551).

Characteristic		*n*	(%)
Sex	Male	261	(47)
	Female	289	(53)
	Not recorded	1	(<1)
Age category	<5	52	(9)
	5–14	136	(25)
	15–24	120	(22)
	25–34	65	(12)
	35–44	77	(14)
	45+	96	(17)
	Not recorded	5	(1)
HIV status	Positive	26	(5)
	Negative	128	(23)
	Not recorded	397	(72)
Facility	Chinhoyi provincial hospital	46	(8)
	Marondera provincial hospital	197	(36)
	Concession district hospital	151	(27)
	Ruwa rehabilitation hospital	99	(18)
	Katanga Utano clinic	28	(5)
	Howard hospital	30	(5)
Relation to index patient	Spouse	68	(12)
	Child	167	(30)
	Guardian	56	(10)
	Sibling	110	(20)
	Another household member	133	(24)
	Not recorded	17	(3)

**Table 4:** TPT regimens and TPT outcomes among HHCs from 157 TB index patients at six health facilities in Zimbabwe—14 November 2023 to 31 July 2024 (*n* = 126).

Characteristics		*n*	(%)
TPT regimen	1HP	48	(40)
	3HP	40	(34)
	3HR	16	(13)
	6H	13	(11)
	6Lfx	2	(2)
	Missing	7	
TPT outcomes	**Successful outcomes**	**111**	**(93%)**
	Completed TPT	78	(70)
	Still on TPT at time of data collection	32	(29)
	Transferred out whilst on TPT	1	(1)
	**Unsuccessful outcomes**	**8**	**(7%)**
	Died	1	(13)
	Not recorded	7	(87)

1HP = 1 month of daily isoniazid and rifapentine; 3HP = 3 months of weekly isoniazid and rifapentine; 3HR = 3 months of daily isoniazid and rifampin; 6H = 6 months of daily isoniazid; 6Lfx = levofloxacin = 6 months of daily levofloxacin. Missing data refers to those recorded as initiated on TPT but where the regimen was not mentioned. These 7 were omitted in the denominator for calculating successful outcomes. Bolded outcome phrase represents summation of specific outcomes below it.

**Table 5:** Timelines for tracing activities among HHCs from 157 TB index patients at six health facilities in Zimbabwe—14 November 2023 to 31 July 2024 (*n* = 126).

Time From	*n* (%)	Total Number	Number with Valid Dates	Median Days (IQR)
Diagnosis of index TB patient to listing HHC		251	237	0 (0–0)
≤7 days	232 (98%)			
>7 days	5 (2%)			
Listing of HHC to reaching clinic		551	313	6 (3–25)
≤7 days	174 (56%)			
8–14 days	32 (10%)			
15–21 days	28 (9%)			
>21 days	79 (25%)			
Listing of HHC to starting TB treatment		12	12	7 (0–10)
≤7 days	8 (73%)			
8–14 days	3 (18%)			
>30 days	1 (9%)			
Listing of HHC to starting TPT		126	107	11 (3–28)
≤7 days	52 (49%)			
8–14 days	10 (9%)			
15–21 days	8 (8%)			
22–30 days	13 (12%)			
>30 days	24 (22%)			

IQR = interquartile range.

## Data Availability

The data presented in this project are available on request from the corresponding author. The data are not publicly available due to authorizations that may be required by the funder.
